# Perceived Neighborhood Crime Safety Moderates the Association Between Racial Discrimination Stress and Chronic Health Conditions Among Hispanic/Latino Adults

**DOI:** 10.3389/fpubh.2021.585157

**Published:** 2021-02-15

**Authors:** Elizabeth L. Budd, Nicole R. Giuliani, Nichole R. Kelly

**Affiliations:** ^1^Counseling Psychology and Human Services, College of Education, University of Oregon, Eugene, OR, United States; ^2^Prevention Science Institute, University of Oregon, Eugene, OR, United States; ^3^Special Education and Clinical Sciences, College of Education, University of Oregon, Eugene, OR, United States

**Keywords:** Hispanic Americans, chronic disease, neighborhood, discrimination, stress

## Abstract

**Background:** Little is known about the link between perceived neighborhood walkability and prevalence of chronic disease. Even less is known regarding this association among Hispanic/Latino adults, despite exhibiting high rates of chronic diseases. Stress due to racial discrimination is a harmful social determinant of health in Hispanics/Latinos. Having both low perceived neighborhood walkability and high racial discrimination stress may exacerbate the chronic disease status of Hispanics/Latinos. Among a U.S. national sample of Hispanic/Latino adults, this cross-sectional study aims to examine (1) the associations among overall perceived neighborhood walkability, racial discrimination stress, and having a chronic health condition; and (2) whether overall perceived neighborhood walkability moderates the hypothesized association between racial discrimination stress and having a chronic health condition.

**Methods:** In January 2018, 798 Hispanic/Latino adults (*M* age = 39.7 years, SD = 15.1; 58.6% female; 70.0% U.S. born; 52.0% Mexican/Mexican American) responded to a survey via Qualtrics Panels. Surveys included the Neighborhood Environment Walkability Scale-Abbreviated, Hispanic Stress Inventory-2, and self-reported presence/absence of chronic health conditions (e.g., hypertension, heart disease). A logistic regression was conducted testing for the moderation of the main effect of racial discrimination stress on the presence of a chronic health condition by overall perceived neighborhood walkability.

**Results:** After controlling for age, body mass index, and income, racial discrimination stress was inversely associated with overall perceived neighborhood walkability (*b* = −0.18, *p* < 0.001) and positively associated with having a chronic health condition (OR = 1.02; 95% CI [1.00, 1.03]). While overall perceived neighborhood walkability was not associated with having a chronic health condition, perceived crime safety was inversely associated with having a chronic health condition (OR = 0.94; 95% CI [0.89, 0.99]). Perceived crime safety moderated the positive association between discrimination stress and having a chronic health condition, such that the association was only significant among those who perceived their neighborhood to be less safe (β = −0.004, 95% CI [−0.01, −0.00]).

**Conclusions:** Overall perceived neighborhood walkability was inversely associated with racial discrimination stress, but not associated with having a chronic health condition. Perceived neighborhood crime safety, but not infrastructure or aesthetics, matters when it comes to the link between racial discrimination stress and having a chronic health condition among Hispanics/Latinos.

## Introduction

In the United States, chronic diseases including cancer, coronary heart disease, and type 2 diabetes are the leading causes of death among Hispanics/Latinos (1, 2). Type 2 diabetes is also more prevalent among Hispanics/Latinos (17%) compared with non-Hispanic Whites (8%) (3). Additionally, Hispanics/Latinos are more likely to develop type 2 diabetes at an earlier age and have more severe diabetes-related complications than the U.S. adult population overall (3).

Neighborhood walkability, which is how conducive the area around one's home is to walking, has been linked to prevalence of chronic disease (4–8). Most work on neighborhood walkability has employed objective measures such as land use, population density, crime rates, and/or proximity to parks (9). A 10-year longitudinal study employing objective measures found that, as neighborhoods became more walkable over time, community-wide diabetes prevalence and cardiovascular disease risk (e.g., high blood pressure and cholesterol) declined (8). Others have reported similar associations using objective measures of neighborhood walkability (4–6). The facilitation of physical activity is one behavioral mechanism identified as connecting greater objectively-measured walkability with lower prevalence of chronic disease (4, 9–12). In comparison, perceived neighborhood walkability is defined as the summation of a person's subjective evaluations of the environment around their home such as their perceptions of aesthetics, built environment features (e.g., presence of crosswalks), and/or safety from crime and traffic (9). A large, multi-country study found certain domains within perceived neighborhood walkability were associated with body mass index (BMI) (7). Most notably, greatest perceived safety from traffic and crime, and nearness to destinations (e.g., stores) were strongly associated with lower BMI (7). The association between perceived neighborhood walkability and chronic diseases is unknown. However, some evidence has suggested that one's perception of neighborhood walkability may have more influence on one's physical activity engagement than objective measures of neighborhood walkability (13, 14).

Furthermore, of the extant literature on the topic, Hispanic/Latino adults make up a small percentage of the overall participants investigated, despite having some of the highest rates of chronic diseases (6, 8, 11). When Hispanics/Latinos are included in studies of neighborhood walkability, they are often compared to their non-Hispanic White peers, hindering our ability to capture within ethnic group variability in experiences, behaviors and health outcomes (15). These data are necessary to inform culturally tailored, community-based interventions to better address disparities in chronic diseases among Hispanic/Latino adults in the United States (16, 17).

A particularly salient social determinant of health among people of color in the United States is racial discrimination (18–20). The Psychosocial Stress Model explains that the stress associated with institutional racism and individual-level experiences with racism lead to health disparities, such as those documented among Hispanic/Latino adults (3, 18, 21). Institutional racial discrimination refers to systems or structural differences that manifest in inequitable distributions of resources and opportunities for people of color. Individual-level experiences of racial discrimination are defined as one's experiences of being treated more poorly than others because of one's race or ethnicity. Empirical evidence suggests that heightened stress due to experiences of racial discrimination is linked to higher rates of chronic diseases by way of several pathways, such as heightened cortisol production, accelerated cellular aging, and poorer health behaviors, including physical activity avoidance, smoking, and greater alcohol consumption (20, 22–24). Meta-analysis results suggest that the inverse association between experiences of racial discrimination and general health may be particularly robust for Hispanic/Latino adults (25). Since experiences of discrimination often take place in public settings (26), including one's neighborhood, it is plausible that perceived neighborhood walkability and racial discrimination stress interact to increase risk for poor health behaviors and associated chronic disease risk. Additionally, neighborhood walkability can be described as an example of institutional racism, such that Hispanics/Latinos disproportionately live in low socioeconomic areas, which is associated with lower objective and perceived neighborhood walkability (27, 28). Theoretical models and empirical literature tend to suggest one's environment is a moderator in the association between psychosocial factors and health/health behaviors (18, 29, 30). However, the association between perceived neighborhood walkability and racial discrimination stress and how they interact in their associations with chronic diseases among Hispanics/Latinos in the United States are unknown.

Among a national sample of U.S. Hispanic/Latino adults, this study aims to (1) test the associations among overall perceived neighborhood walkability, racial discrimination stress, and having a chronic health condition; and (2) examine whether overall perceived neighborhood walkability moderates the association between racial discrimination stress and having a chronic health condition.

Informed by the aforementioned literature, hypotheses to the study aims are outlined as follows. Regarding aim 1, it is hypothesized that there will be a significant inverse association between racial discrimination stress and overall perceived neighborhood walkability (26, 28, 31); a significant positive association between racial discrimination stress and having a chronic health condition (18, 20, 22–25); and a significant inverse association between overall perceived neighborhood walkability and having a chronic health condition (7, 11). Regarding aim 2, it is hypothesized that overall perceived neighborhood walkability will significantly moderate the association between racial discrimination stress and having a chronic health condition (18, 29, 30), such that higher overall perceived neighborhood walkability will reduce the strength of the association between racial discrimination stress and having a chronic health condition, adjusting for age, BMI, annual household income, acculturation, U.S. region of residence, and health insurance status (32–41).

The findings of this study advance the literature by elucidating the associations among social and environmental factors and the presence of a chronic health condition among Hispanic/Latino adults, a sizable sub-set of the U.S. population at heightened risk for chronic diseases (3, 21). A better understanding of these associations could inform culturally tailored, community-based strategies to better promote healthy behaviors and prevent chronic diseases among Hispanics/Latinos in the United States (16, 17, 42).

## Materials and Methods

### Data Collection

In January 2018, Qualtrics Panels was used to recruit Hispanic/Latino adults to complete a 197-question electronic survey in English or Spanish, which assessed their demographic information, health behaviors, physical and mental health conditions, and perceptions of their social and physical environments. Measures in the survey that were not already validated in Spanish were translated forward and backward to Spanish from English by two bi-lingual research assistants. Eligible respondents were 18 years or older, identified as Hispanic or Latino, resided in the United States, and were fluent in English or Spanish. Qualtrics Panels partners with over 20 online panel providers, namely market research panels, to supply a nationwide sample of potential participants who fit the eligibility requirements of this cross-sectional study (43).

Potential research participants were contacted by Qualtrics Panels via an email invitation. To avoid self-selection bias, the specific details of the study were not included in the email invitation. Those who expressed interest in participating were sent study details and underwent informed consent protocols in line with the committee responsible for human experimentation (institutional and national) and with the World Medical Association's Declaration of Helsinki. Specifically, prior to taking the survey, all participants provided their consent by selecting a radial button that read, “I have read the contents of this consent form. I give my consent to participate in this study,” which appeared at the bottom of the electronic informed consent form. Written documentation of consent was waived. Participants who completed the survey, received a pre-determined amount of points (stated in the email invitation to participate in the study). The amount of points was determined by the length of the survey, participant's specific panelist profile, and target acquisition difficulty. The points earned for completion of the survey can be redeemed for cash, airline miles, gift cards, points on online games, sweepstakes entrances and vouchers. This research protocol was approved by the Institutional Review Board at the University of Oregon. The dataset analyzed for this study can be found in the Harvard Dataverse, (44).

### Measures

Demographic characteristics of the respondents were assessed using the Demographic and Health Information Data Questionnaire (45, 46). Demographic characteristics reported in this study include respondents' age, height, weight, gender, ethnic origin, whether English was their first language, nativity, acculturation, highest level of education, employment status, U.S. region of residence, relationship status, health insurance status, and annual household income. Response options for all demographic characteristics appear in Table 1. BMI was calculated using the following equation: 703 × weight (lbs) /[height (in)] ∧ 2 (47). Seven respondents were excluded from the analyses because of seemingly implausible BMI values (<12 or >65). Annual household income was assessed by asking respondents to select the total combined income that is made yearly by all working members of their household from 11 U.S. dollar ranges. Response options were collapsed into: <$10,000–$29,000, $30,000–$69,999, and $70,000–$100,000+. Acculturation was assessed with The Abbreviated Multidimensional Acculturation Scale, which includes 6 U.S. cultural identity items (e.g., “I think of myself as being U.S. American”), nine English language competence items (e.g., “How well do you speak English on the phone?”), and six U.S. cultural competence items (e.g., “How well do you know popular American television shows?”) (48). U.S. cultural identity items were assessed on a Four-point-Likert scale with response options ranging from 1 (“strongly disagree”) to 4 (“strongly agree”). English language competence and U.S. cultural competence were assessed on a Four-point-Likert scale with response options ranging from 1 (“not at all”) to 4 (“extremely well”). The Abbreviated Multidimensional Acculturation Scale has been shown to produce reliable and valid responses among Hispanics/Latinos (48). A mean was calculated to create a total U.S. acculturation score from all of the items within the U.S. cultural identity, English language competence, and U.S. cultural competence sub-scales. A higher mean score denotes a greater degree of acculturation to the United States.

**Table 1:** Descriptive characteristics of the study sample and key study variables.

**Variable**	***N***	**Missing**	**Mean**	**Skewness**	**Kurtosis**	**Min.**,
			**(S.D.)^a^**	**(S.E.)^b^**	**(S.E.)**	**Max**.
Age	797	1	39.65	0.307	−0.955	18, 81
			15.05	0.087	0.173	
Body mass	774	24	28.3	1.06	1.72	13.70
index			7.266	0.088	0.176	64.19
Acculturation	791	7	71.46	−1.18	1.83	21, 84
			10.59	0.087	0.174	
Perceived	794	4	46.88	−0.184	0.014	21, 64
neighborhood walkability			7.25	0.087	0.173	
Racial	789	9	14.237	0.937	0.35	0, 55
discrimination stress			12.67	0.087	0.174	
**Variable**					***N*** **(Valid %)**	
Gender						
Woman					467 (58.6)	
Man					320 (40.2)	
Transgender					8 (1.0)	
Other					2 (0.3)	
Missing					1	
Ethnic origin						
Mexican or Mexican American					415 (52.0)	
Puerto Rican					136 (17.0)	
Cuban					68 (8.5)	
Spanish					44 (5.5)	
Dominican					27 (3.4)	
Another Hispanic or Latino origin (e.g., Columbian, Venezuelan, Peruvian)					108 (13.5)	
Missing					0	
English is first language						
Yes					522 (65.4)	
No					276 (34.6)	
Missing					0	
Nativity						
U.S. born					556 (70.0)	
Born outside of the United States					238 (30.0)	
Missing					4	
Highest level of education completed						
Less than high school					22 (2.8)	
High school or GED					217 (27.6)	
Trade/Technical school					60 (7.6)	
2-year college					162 (20.6)	
4-year college/university					237 (30.2)	
Professional/Graduate					88 (11.2)	
Missing					12	
Employment status						
Work full-time					395 (49.7)	
Work part-time					114 (14.3)	
Unemployed but looking for a job					97 (12.2)	
Do not work (e.g., stay-at home parent, retired, on disability, etc.)					189 (23.8)	
Missing					3	
Relationship status						
I am married or in a civil union					364 (45.6)	
I am single, I do not have a spouse or partner					284 (35.6)	
I am not married, but in a relationship					132 (16.5)	
I am widowed					18 (2.3)	
Missing					0	
U.S. region of residence						
South					316 (39.6)	
West					182 (22.8)	
Northeast					155 (19.4)	
Midwest					145 (18.2)	
Missing					0	
Health insurance status						
Private insurance (e.g., HMO, PPO)					392 (49.1)	
Public insurance (e.g., Medicare, Medicaid)					304 (38.1)	
Uninsured					102 (12.8)	
Missing					0	
Annual household income						
<$10,000–29,000					231 (29.0)	
$30,000–69,999					300 (37.6)	
$70,000–100,000+					266 (33.4)	
Missing					1	
Current chronic health conditions reported						
High cholesterol					175 (21.9)^c^	
Heart disease					27 (3.4)	
Cancer					15 (1.9)	
Hypertension					181 (22.7)	
Type 2 diabetes					122 (15.3)	
None					54 (6.8)	
Missing					0	
Presence of a chronic health condition						
At least one current chronic health condition					362 (45.4)	
No current chronic health conditions					436 (54.6)	
Missing					0	

a S.D.: standard deviation;

b S.E.: standard error;

c *Respondents could report having more than one chronic health condition, thus, percentages reflect the proportion of the sample that endorsed having each of the listed chronic health conditions*.

The independent variable, racial discrimination stress, was measured by the total score of the Discrimination Stress sub-scale (11 items; e.g., “I was discriminated against because of my customs and cultural celebrations”) from the Hispanic Stress Inventory-2. The Discrimination Stress sub-scale assesses psychosocial stress from discriminatory experiences related to being Hispanic. For each item, respondents were asked if they have experienced the reported stressor (Yes/No), and if they had experienced the stressor, they were asked to rate how stressful the event was on a five-point-Likert scale ranging from 1 (“not at all worried/tense”) to 5 (“extremely worried/tense”). Possible scores ranged from zero to 55 and higher scores indicate greater racial discrimination stress. The Hispanic Stress Inventory-2 sub-scales have been shown to produce reliable and valid responses in Spanish and English among a diverse sample of Hispanic/Latino adults in the United States (49, 50). In this sample, the Discrimination Stress sub-scale had good reliability (α = .933).

The dependent variable, presence of a chronic health condition, was assessed by one question from the Demographic and Health Information Data Questionnaire (45, 46). Respondents were asked to indicate all of the health conditions they currently have from the following: high cholesterol, overweight/obesity, heart disease, cancer (specify the type), high blood pressure, type 2 diabetes, other (specify), or to select “I do not have any of these health conditions.”

The hypothesized moderator, overall perceived neighborhood walkability, was measured using a total sum score of four sub-scale scores from the Neighborhood Environment Walkability Scale- Abbreviated (NEWS-A) including: infrastructure for walking/cycling (six items; e.g., “There are sidewalks on most of the streets in my neighborhood”), aesthetics (four items; e.g., “There are trees along the streets in my neighborhood”), traffic safety (three items; e.g., “The speed of traffic on most *nearby* streets is usually slow (30 mph or less)”), and crime safety (three items; e.g., “The crime rate in my neighborhood makes it unsafe to go on walks during the day”) (51). Respondents chose from four-point Likert scale response options ranging from 1 (“strongly disagree”) to 4 (“strongly agree”) for each item. Five items were reverse coded to align with the direction of the other items. Possible scores ranged from 16 to 64 and higher scores indicate greater overall perceived neighborhood walkability. The scale has demonstrated validity and reliability in English and Spanish (52, 53). In this sample, the overall NEWS-A had adequate reliability (α = 0.761).

### Analyses

Descriptive statistics were conducted to review missing cases and response distributions for scale variables with a skew or kurtosis outside of ±2 (54). Preliminary analyses also included a Pearson correlation in order to examine potential multicollinearity (*r* > ± 0.80) between key study variables and independent-samples *t*-tests to assess unadjusted associations between key study variables and the dependent variable (55).

Regarding the dependent variable, having “overweight/obesity” was removed as one of the chronic conditions. Instead, BMI was included as a covariate in the regression models because of its association with chronic disease risk (39). Respondents endorsed having up to four of the six included chronic health conditions. Of those who endorsed having at least one of the health conditions, 60.22% reported having only one of the conditions followed by 23.48% with two. Given this distribution, responses were dichotomized into whether the respondent endorsed currently having either any or none of the health conditions. Prior research also supports treating presence of a chronic health condition as a binary term (36, 56), given meaningful differences between the two groups in health service use (e.g., emergency room visits) and general medical expenditures (36).

To address study aim 1, one multiple linear regression model and two binary logistic regression models were conducted. Age, annual household income, BMI, acculturation, U.S. region of residence, and health insurance status were initially included in these models as covariates because of their associations with having a chronic health condition (32–41). In pursuit of parsimonious models, covariates were removed that had non-significant (*p* ≥ 0.05) regression coefficients in all of the regression models conducted for study aims 1 and 2, and also had no notable effects on the models' variance explained or results when they were removed (57). First, the association between overall perceived neighborhood walkability and racial discrimination stress, adjusting for the covariates, was assessed in a multiple linear regression model. Second, the main effects of racial discrimination stress on presence of a chronic health condition, adjusting for the covariates, was investigated using binary logistic regression. Third, the main effects of overall perceived neighborhood walkability on the presence of a chronic health condition, adjusting for the covariates, was conducted using binary logistic regression. The binomial assumption was assumed because there was no reason to believe there was dependence among observations or that the sample was non-random (58).

For study aim 2, overall perceived neighborhood walkability and racial discrimination stress were grand-mean centered and an interaction term was created. A binary logistic model was conducted that included: the covariates, centered overall perceived neighborhood walkability, centered racial discrimination stress, and the interaction term. All statistical analyses were conducted in IBM SPSS Statistics for Windows, Version 26 (59). The moderation plot was conducted in R, Version 3.6.1 using the interactions package (60, 61).

### Exploratory Analyses

For *post hoc* exploratory purposes, aims 1 and 2 were re-examined using the individual sub-scales of the NEWS-A. Infrastructure for walking/cycling (α = 0.764), aesthetics (α = 0.799), and crime safety (α = 0.912) demonstrated adequate to good reliability in this sample. Traffic safety demonstrated poor reliability (α = 0.258) in this sample and was thus not included in the exploratory analyses. Preliminarily, Pearson correlations were conducted to assess associations among the three sub-scales. The same statistical procedures were conducted as described earlier for the study aims, except they were repeated three times, once for each NEWS-A sub-scale.

## Results

### Sample

The survey respondents included 798 Hispanic/Latino adults 18–81 years old (*M* = 39.7 ± 15.1 years; BMI *M* = 28.30 ± 7.3 kg/m^2^; Table 1). Approximately 20% of respondents completed the survey in Spanish, 80% in English. The majority of the sample identified as women (58.6%); Mexican or Mexican American (52%); native English speakers (65.4%); U.S. born (70.0%); educated through 2 years of college or beyond (62%); and employed at least part time (64%). Respondents resided in all major regions of the United States, with the largest portion living in the South (39.6%). Additionally, the largest proportions of respondents had a least one chronic health condition (45.4%); were married or in a civil union (45.6%); had public or private health insurance (87.2%); and an annual household income of $30,000–69,999 (37.6%).

### Preliminary Findings

In unadjusted associations, there was a significant inverse association between overall perceived neighborhood walkability and racial discrimination stress (*r* = −0.166, *p* < 0.01). The mean scores of overall perceived neighborhood walkability (*t*_(787)_ = −0.695, *p* = 0.487) and racial discrimination stress (*t*_(792)_ = −0.963, *p* = 0.336) were not significantly different for those with or without a chronic health condition.

### Study Aim 1 Results

The covariates retained in aim 1 and aim 2 regression models were age, BMI, and annual household income. There was a significant and inverse association between overall perceived neighborhood walkability and racial discrimination stress (*b* = −0.18, *p* < 0.001; *R*^2^ = 0.07).

Table 2 provides an overview of results from the logistic regression analyses. In Model 1, there was a significant and positive association between racial discrimination stress and the presence of a chronic health condition (Odds Ratio [OR] = 1.017; 95% Confidence Interval [CI] [1.004, 1.030]). In Model 2, there was no significant association between overall perceived neighborhood walkability and the presence of a chronic health condition (OR = 0.993; 95% CI [0.72, 1.015]).

**Table 2:** Logistic regression results for predicting presence of a chronic health condition among U.S. Hispanic/Latino adults.

**Variables**	**Model 1**		**Model 2**		**Model 3**	
	**Odds ratio^a^**	**95% Confidence**	**Odds ratio**	**95% Confidence**	**Odds ratio**	**95% Confidence**
		**interval**		**interval**		**interval**
Age	1.047^***^	1.036–1.059	1.044^***^	1.033–1.056	1.047^***^	1.035–1.059
Annual household income						
<$10,000–$29,000	ref		ref		ref	
$30,000–$69,999	1.396	0.950–2.051	1.452	0.992–2.126	1.417	0.963–2.084
$70,000–$100,000+	1.697^**^	1.139–2.527	1.763^**^	1.185–2.622	1.721^**^	1.152–2.572
Body mass index	1.046^***^	1.023–1.069	1.047^***^	1.024–1.070	1.046^***^	1.023–1.069
Racial discrimination stress	1.017^*^	1.004–1.030	N/A		1.016^*^	1.003–1.029
Perceived neighborhood walkability	N/A^c^		0.993	0.972–1.015	0.996	0.974–1.018
Perceived neighborhood walkability X Racial discrimination stress	N/A		N/A		0.999	0.997–1.001
**Model diagnostics**						
−2 Log likelihood	944.713		957.788		941.563	
Omnibus model chi-square	108.825^***^		104.122^***^		109.184^***^	
Degrees of freedom	5		5		7	
Hosmer and Lemeshow chi-square	15.937^*^		11.579		12.259	
Degrees of freedom	8		8		8	
Nagelkerke R-squared	0.177		0.169		0.178	

a Odds ratios for the constant not displayed in the table;

b ref, reference group;

c N/A, variable is not applicable to the model;

d Model 3 includes grand-mean centered variables: perceived neighborhood walkability, racial discrimination stress, and the interaction term;

* p < 0.05,

** p < 0.010,

*** *p < 0.001 (two-tailed)*.

### Study Aim 2 Results

The full model results displayed in Model 3 within Table 2 show that the interaction between racial discrimination stress and perceived walkability on having a chronic health condition was not significant (OR = 0.999; 95% CI [0.997, 1.001]), suggesting the association between racial discrimination stress and health did not vary by overall perceived neighborhood walkability. Model 3 explained a total 17.8% of the variance in the presence of a chronic health condition.

### Exploratory Results

Unadjusted bivariate associations among the three NEWS-A sub-scales showed crime safety was inversely associated with infrastructure for walking/cycling (*r* = −0.106, *p* < 0.01) and not significantly associated with aesthetics (*r* = 0.059, *p* = 0.09). Infrastructure for walking/cycling was positively associated with aesthetics (*r* = 0.520, *p* < 0.01).

Table 3 shows the association between each NEWS-A sub-scale and racial discrimination stress, adjusted for age, BMI, and annual household income. Only crime safety was significantly and inversely associated with racial discrimination stress (*b* = −0.27, *p* < 0.001).

**Table 3:** Adjusted associations between each Neighborhood Environment Walkability Scale- abbreviated sub-scale and racial discrimination stress among U.S. Hispanic/Latino adults.

**Variable**	***b***	**95% Confidence interval**	**S.E.^a^**	***t***	**df^b^**	***p***	***R*^2^**
Infrastructure for walking/cycling	−0.030	−0.211–0.086	0.076	−0.827	793	0.408	0.033
Aesthetics	−0.036	−0.314–0.103	0.106	−0.991	793	0.322	0.033
Crime safety	−0.265	−0.959−0.595	0.102	−7.472	793	<0.001	0.099

a S.E.: standard error;

b df: degrees of freedom;

c *Covariates in the multiple linear regression models include age, body mass index, and annual household income*.

**Figure 1:**
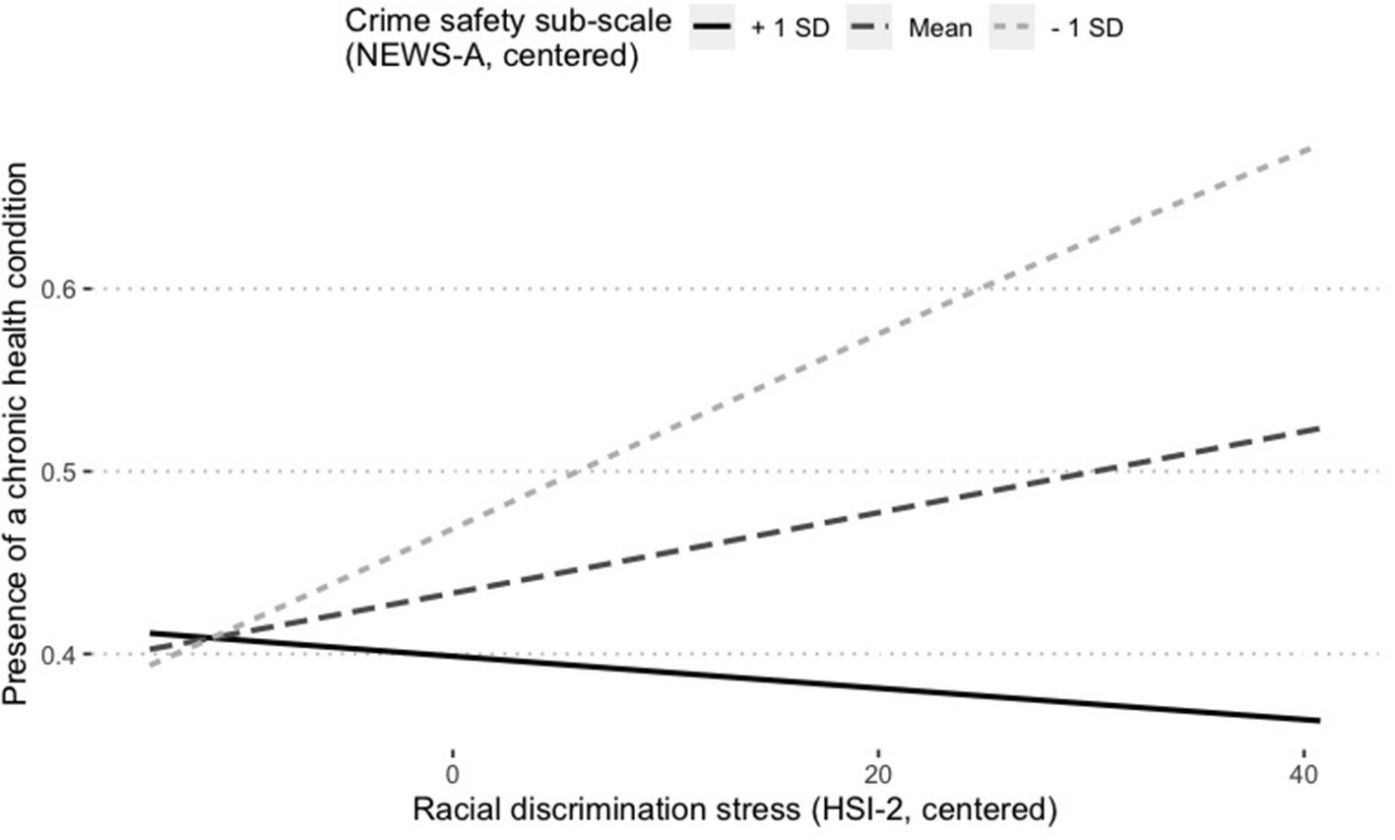
Perceived crime safety moderates the association between racial discrimination stress and having a chronic health condition among U.S. Hispanic/Latino adults. NEWS-A, Neighborhood Environment Walkability Scale-Abbreviated; HSI-2, Hispanic Stress Inventory-2; SD, standard deviation. Simple slopes indicate the positive association between racial discrimination stress and presence of a chronic health condition is significant only among those who report lower perceived neighborhood safety from crime (β = 0.02, *p* = 0.01).

Among the three NEWS-A sub-scales, there was only a significant and inverse association between crime safety and the presence of a chronic health condition (OR = 0.936; 95% CI [0.887, 0.988]; Table 4). There was also a significant moderating effect of crime safety on the association between racial discrimination stress and presence of a chronic health condition. Specifically, there was a significant and positive association between discrimination stress and having a chronic health condition, only among those who perceived their neighborhood to be less safe (β = −0.0043, SE = 0.0021, CI[−0.0084, −0.0002], *p* < 0.05; Figure 1).

**Table 4:** Predicting presence of a chronic health condition and testing for moderating effects of perceived neighborhood walkability sub-scales among U.S. Hispanic/Latino adults.

**Variables**	**Main effects models**		**Moderating models**	
	**Odds ratio^a^**	**95% Confidence**	**Odds ratio**	**95% Confidence**
		**interval**		**interval**
**PERCEIVED INFRASTRUCTURE FOR WALKING/CYCLING SUB-SCALE**				
Age	1.044^***^	1.033–1.055	1.047	1.036–1.059
Body mass index	1.048^***^	1.025–1.071	1.046^***^	1.023–1.070
Annual household income						
< $10,000–$29,000	ref		ref	
$30,000–$69,999	1.424	0.974–2.082	1.397	0.950–2.053
$70,000–$100,000+	1.700^**^	1.025–2.524	1.1670^*^	1.117–2.497
Racial discrimination stress	N/A^c^		1.017^*^	1.004–1.030
Perceived infrastructure for walking/cycling	1.014	0.975–1.054	1.012	0.973–1.053
Perceived infrastructure for walking/cycling X Racial discrimination stress	N/A		1.000	0.997–1.004
**Model diagnostics**						
−2 Log likelihood	960.059		944.350	
Omnibus model chi-square	104.642^***^		109.189^***^	
Degrees of freedom	5		7	
Hosmer and Lemeshow chi-square	12.978		17.507^*^	
Degrees of freedom	8		8	
Nagelkerke R-squared	0.169		0.178	
**PERCEIVED AESTHETICS SUB-SCALE**				
Age	1.043^***^	1.032–1.054	1.047^***^	1.064–1.059
Body mass index	1.048^***^	1.025–1.071	1.046^***^	1.023–1.069
Annual household income						
<$10,000–$29,000	ref		ref	
$30,000–$69,999	1.428	0.976–2.088	1.413	0.961–2.078
$70,000–$100,000+	1.704^**^	1.147–2.531	1.674^*^	1.121–2.501
Racial discrimination stress	N/A		1.016^*^	1.004–1.029
Perceived aesthetics	1.022	0.968–1.079	1.021	0.967–1.078
Perceived aesthetics X Racial discrimination stress	N/A		1.003	0.998–1.007
**Model diagnostics**						
−2 Log likelihood	959.108		942.063	
Omnibus model chi-square	104.007^***^		109.887^***^	
Degrees of freedom	5		7	
Hosmer and Lemeshow chi-square	12.495		23.841	
Degrees of freedom	8		8	
Nagelkerke R-squared	0.169		0.179	
**PERCEIVED CRIME SAFETY SUB-SCALE**				
Age	1.047^***^	1.036–1.059	1.049^***^	1.037–1.061
Body mass index	1.048^***^	1.025–1.071	1.046^***^	1.023–2.455
Annual household income						
<$10,000–$29,000	ref		ref	
$30,000–$69,999	1.477^*^	1.007–2.166	1.426	0.967–2.103
$70,000–$100,000+	1.731^**^	1.167–2.166	1.644^*^	1.101–2.455
Racial discrimination stress	N/A		1.009	0.995–1.023
Perceived crime safety	0.936^*^	0.887–0.988	0.951	0.899–1.007
Perceived crime safety X Racial discrimination stress	N/A		0.996^*^	0.992–0.999
**Model diagnostics**						
−2 Log likelihood	953.214		935.718	
Omnibus model chi-square	110.280^***^		116.615^***^	
Degrees of freedom	5		7	
Hosmer and Lemeshow chi-square	8.952		10.304	
Degrees of freedom	8		8	
Nagelkerke R-squared	0.178		0.189	

a Odds ratios for the constant not displayed in the table;

b ref, reference group;

c N/A, variable is not applicable to the model;

d The models testing for moderation include grand-mean centered variables for perceived neighborhood walkability sub-scales, perceived racial discrimination stress, and the interaction terms;

* p < 0.05,

** p < 0.010,

*** *p < 0.001 (two-tailed)*.

## Discussion

The present study examined (1) the associations among overall perceived neighborhood walkability, racial discrimination stress, and having a chronic health condition; and (2) whether overall perceived neighborhood walkability moderated the hypothesized association between racial discrimination stress and having a chronic health condition among a U.S. national sample of Hispanic/Latino adults. Study findings advance the understanding of how experiences such as, how individuals perceive the safety of their neighborhoods and the degree to which individuals experience racial discrimination stress, vary among Hispanics/Latinos in the United States and how these experiences are associated with having a chronic health condition. The study findings can inform next steps in research and practice that aim to eliminate current chronic disease disparities among Hispanic/Latino adults and details for which are provided below (3, 21).

As hypothesized, those who reported experiencing more racial discrimination stress had higher odds of having a chronic health condition after adjusting for age, BMI, and annual household income. This finding is consistent with The Psychological Stress Model (18) and extant literature among Hispanics/Latinos and other racial/ethnic miniories (19, 23, 25, 49). While the increased odds for having a chronic health condition among those who report more racial discrimination stress was marginal in this study, alongside the extant literature, the finding reinforces the importance of reducing racial discrimination against Hispanics/Latinos in the United States in order to promote health equity.

Furthermore, as hypothesized (26, 28, 31), an inverse association, although weak, was found between overall perceived neighborhood walkability and racial discrimination stress. Overall perceived neighborhood walkability is a total of four individual sub-scales. Exploratory results indicated perceived safety from crime, not perceived infrastructure for walking/cycling and aesthetics of one's neighborhood, was the driver of the inverse association found between overall perceived neighborhood walkability and racial discrimination stress. These findings not only advance our understanding of the association between these environmental and social factors among U.S. Hispanic/Latino adults, they are also novel to the broader literature not focused on Hispanics/Latinos. In related literature, a systematic review found consistent support for the inverse association between objectively measured neighborhood walkability and general psychosocial distress (62). In addition to focusing on objective vs. perceived neighborhood walkability, the reviewed studies did not assess racial discrimination stress specifically and only one of the studies, by Brown and colleagues (63), was strictly among U.S. Hispanic/Latino adults (62, 63). Brown et al.'s (63) study involved low socioeconomic, U.S. Hispanic/Latino, older adults and found that front porches were positively associated with social support, which was associated with lower psychological distress (63). The presence of front porches is most similar to items in the infrastructure for walking/cycling sub-scale within the NEWS-A. This association between infrastructure for walking/cycling and racial discrimination stress was not identified in the present study, which could be due to the use of different measures of perceived neighborhood walkability between the studies and substantial demographic differences between the study samples. Future research could investigate social support and other potential links connecting perceived neighborhood crime safety and racial discrimination stress among Hispanic/Latino adults.

Contrary to the hypothesis, overall perceived neighborhood walkability was not associated with having a chronic health condition. However, exploratory findings showed that perceived neighborhood safety from crime was inversely associated with having a chronic health condition. Both of these findings contribute toward filling the gap in knowledge regarding perceived (vs. objective) neighborhood walkability and chronic diseases (4–6, 8). There is a precedent for perceived neighborhood safety playing a pivotal role in behavioral and health risk factors for chronic diseases in the literature. For example, another study of Hispanics/Latinos found infrastructure for walking/cycling was positively associated with physical activity, but only when perceived crime was low (64). Also, a multi-country study by De Bourdeaudhuij and colleagues (7) found perceived neighborhood safety from traffic and crime, and nearness to destinations were inversely associated with BMI, adjusting for relevant demographic factors (7). Where BMI was the dependent variable in De Bourdeaudhuij's (7) study, BMI was a covariate in the present study. The latter approach is advantageous when aiming to reduce chronic disease rates among Hispanics/Latinos, as higher BMI is just one of many risk factors for chronic diseases (65).

Overall perceived neighborhood walkability did not moderate the association between racial discrimination stress and presence of a chronic health condition, contrary to the hypothesis. However, exploratory findings did identify a significant moderating effect of perceived crime safety on the association between racial discrimination stress and presence of a chronic health condition. Specifically, the positive association between racial discrimination stress and presence of a chronic health condition is significant only among those who report lower perceived neighborhood safety from crime. This finding is consistent with theoretical models and empirical literature that have characterized the environment as a moderator of the association between psychosocial factors and health/health behaviors (18, 29, 30). The findings also highlight the importance of studying and targeting the perceived walkability of neighborhoods in more specific rather than general ways. Examining the study aims by each NEWS-A sub-scale brought clarity where overall perceived neighborhood walkability could not.

This study's findings suggest perceived neighborhood safety matters, not infrastructure and aesthetics, when it comes to racial discrimination stress and having a chronic health condition among Hispanics/Latinos. Low perceived neighborhood safety from crime could reflect institutional racism, as Hispanics/Latinos are more likely than non-Hispanic Whites to be victims of violent crimes and more likely to live in poverty, which is associated with higher crime rates (66, 67). Improving safety in communities where Hispanic/Latino residents report greater safety concerns may have beneficial effects on racial discrimination stress and health among Hispanic/Latino adults. Testing such an intervention in a longitudinal study is a recommended next step in research.

This study responds to calls in the literature to investigate innovative explanations for health disparities by testing interactions of the physical and social environments (68, 69). A study that examined the moderating role of the environment on racial discrimination stress and health among African Americans found that discrimination experienced in the previous year was positively associated with cortisol concentration among those residing in neighborhoods with more White residents (69). Building on the present study's findings by including an additional aspect of the social environment, such as social capital (70), perceived neighborhood social cohesion (71), or neighborhood racial makeup (69), may explain more of the total variance in presence of a chronic health condition among Hispanic/Latino adults.

The study sample reflected the Hispanic/Latino population in the United States well on a number of sociodemographic characteristics including proportions by ethnic origins, nativity, employment status, relationship status, and health insurance status (72, 73). The sample appears to over represent women (sample 58% vs. U.S. 49%) and those who have completed 2 years or more of college (sample 62% vs. U.S. 41%), and underrepresent those living in the Western region of the United States (sample 23% vs. 41%) (73, 74). These disproportionate representations in the sample could have implications for the findings, but explanations follow that propose the implications may be minimal. Suspecting the study results could vary by gender, *post hoc* analyses were conducted and the study findings did not change when examined by women and men. Additionally, those with more education are more likely to have a primary healthcare provider and more likely to effectively manage chronic health conditions, like hypertension, than those less educated (75, 76). Thus, this study's findings could reflect the experiences of a healthier group of Hispanics/Latinos than the average national cross-section of Hispanics/Latinos. A RAND Corporation report on nationally representative data from the Medical Expenditure Panel Survey from 2014 shows that 49% of Hispanic/Latino adults in the United States have at least one chronic health condition. This is 3.6% more than respondents endorsed in this study, and the two studies generally assessed the same conditions. Finally, the Western region of the United States, California in particular, is home to the largest proportion of the U.S. Hispanic/Latino population. One national survey found that the West generally fares better on indices of residential, racial integration than other U.S. regions, but the state of race relations between non-Hispanic Whites and Hispanic/Latinos were similar to the rest of the country (77). Region was also included in the initial analyses as a covariate and removed only after it was determined to not be an explanatory contributor to the models.

### Strengths and Limitations

The most notable strengths of this study are the several novel findings that advance the literature and inform next steps in practice and research that aim to address health disparities among Hispanic/Latino adults in the United States. Additional study strengths are that the survey was provided in English and Spanish and the sample reflected the U.S. Hispanic/Latino population well on a number of characteristics; both increase generalizability of the study results. A large sample size of Hispanic/Latino adults provides ample power to test for moderation, which is often underpowered in the literature (78), and allows for the examination of within ethnic group variability in experiences and chronic health conditions.

Lastly, there are also study limitations. The cross-sectional design limits the study interpretations to associations rather than causal inferences. The inability to measure survey response rate using Qualtrics Panels and the un-measured differences between those who are more and less likely to participate in electronically delivered surveys makes the study vulnerable to selection bias. Although, a meta-analysis by Walter and colleagues (79) did find similar internal reliability and external validity between online panel and other sampling techniques (79). The self-report measure of current chronic health conditions, as opposed to medically confirmed, likely underestimates the actual proportion of those who have chronic health conditions, as undiagnosed type 2 diabetes is common, especially among people of color in the United States (80). Future longitudinal studies should focus on disentangling mechanisms by chronic condition, as some mechanisms are shared and others are not. Finally, the racial discrimination stress measure was not specific to racial discrimination experienced in one's neighborhood, however, it does capture stress due to racial discrimination in the contexts of one's daily life, which would include the neighborhood in which one lives.

## Conclusion

Novel insights of this study include the associations among perceived neighborhood walkability, racial discrimination stress, and having a chronic health condition among U.S. Hispanic/Latino adults, as well as evidence that perceived neighborhood safety plays a moderating role in the positive association between racial discrimination stress and having a chronic health condition. Improving safety in communities where Hispanic/Latino residents report greater safety concerns may have beneficial effects on racial discrimination stress and health among Hispanic/Latino adults.

## Data Availability Statement

The dataset analyzed for this study can be found in the Harvard Dataverse, https://doi.org/10.7910/DVN/NABLZX.

## Ethics Statement

The study involving human participants was reviewed and approved by Institutional Review Board at the University of Oregon. Written informed consent for participation was not required for this study in accordance with the national legislation and the institutional requirements.

## Author Contributions

EB contributed to the conception and design of the study, analysis and interpretation of data, and drafting of the full manuscript. NG and NK contributed to the conception and design of the study, analysis and interpretation of data, and manuscript revisions. All authors read and approved the submitted version.

## Conflict of Interest

The authors declare that the research was conducted in the absence of any commercial or financial relationships that could be construed as a potential conflict of interest.
